# Induction of Viable but Nonculturable *Escherichia coli* O157:H7 by Low Temperature and Its Resuscitation

**DOI:** 10.3389/fmicb.2018.02728

**Published:** 2018-11-13

**Authors:** Caijiao Wei, Xihong Zhao

**Affiliations:** Research Center for Environmental Ecology and Engineering, Key Laboratory for Green Chemical Process of Ministry of Education, Key Laboratory for Hubei Novel Reactor and Green Chemical Technology, School of Environmental Ecology and Biological Engineering, Wuhan Institute of Technology, Wuhan, China

**Keywords:** viable but non-culturable state, *Escherichia coli* O157:H7, resuscitation, low temperature, induction, food safety

## Abstract

Viable but non-culturable (VBNC) cells are alive bacteria cells, but lose their culturability in conventional culture media, usually escape detection by the plate count method and pose a serious threat to food safety and public health. Therefore, it is urgent to study the VBNC status, and to provide theoretical basis and scientific basis for food processing and safety control caused by pathogenic microorganisms. In this study, *Escherichia coli* O157:H7 was induced to the VBNC state at two different temperatures (-20°C and 4°C) and its resuscitation and morphological changes under different nutritional conditions were studied. The initial inoculum of 2.1 × 10^7^ CFU/mL *E. coli* O157:H7 cells were induced into the VBNC state in normal saline, distilled water, LB broth at -20 °C after 176, 160, 80 days, respectively. The results showed that *E. coli* O157:H7 reserved at -20°C, and LB culture medium were easier to enter VBNC state than others conditions, the cells still had metabolic activity and the cell morphology changed from the typical rod shape to short rod and the cell size decreased. The resuscitate ways including the direct warming resuscitation, gradual warming resuscitation, adding chemical substance resuscitation, and adding nutrients resuscitation were studied. The results showed that the optimal conditions of 5% Tween 80 and 3% Tween 80 acculated the resuscitation of *E. coli* O157:H7 VBNC state cells induced by low temperature LB medium and low temperature saline. *E. coli* O157:H7 VBNC state failed from resuscitation when incubating in LB broth, respectively using direct warming and adding nutrients substance. This study provides new insights into induction and resuscitation of VBNC *E. coli* O157:H7 and offers an approach for investigating the formation mechanism of VBNC foodborne pathogens in food safety.

## Introduction

Foodborne pathogens are tremendous threats to food safety and global health issues, which can cause severe disease in humans via contaminated water or food. According to the data from the World Health Organization, there are many outbreaks and numerous sporadic death cases per year caused by *Escherichia coli* O157:H7, *Staphylococcus aureus, Salmonella enterica, Listeria monocytogenes, Campylobacter jejuni*, and so on (Zhao et al., 2014). For example, in 2011, an outbreak of caused by *E. coli* O104:H4 in Germany brought enormous effect on social, political, and economic implications throughout the whole Europe. In particular, *E. coli* O157:H7 is recognized internationally as one of the most significant foodborne pathogens, because it can cause abdominal pain, diarrhea, and more serious complications with a low infectious dose (as few as 10 cells), such as hemorrhagic enteritis, hemolytic uremic syndrome, and thrombotic thrombocytopenic purpura (Zhao et al., 2010; Mcleod et al., 2016). Additionally, *E. coli* O157:H7 has several other modes of survival, which includes viable but non-culturable (VBNC) state and formation of biofilms and so on (Li et al., 2014).

The VBNC state was first discovered and introduced by Xu et al. (1982). When conditions are unsuitable for sustaining normal growth, this state is a survival strategy by many bacteria. In this physiological condition, the cells that could not form colony on enriched agar media, but exhibit detectable metabolic function. The bacteria in such a state show a discrete metabolic activity, but are not able to replicate. There are many factors that caused bacteria to enter VBNC states, such as temperature, salinity, pH, nutrient limitation, humidity, and so on. To date, researchers have identified 67 species of pathogenic bacteria that can enter the VBNC state (Zhao et al., 2017). However, some of them can retain virulence or resuscitate into culturable cells under certain conditions. Oliver et al. (1995) found that VBNC *Vibrio vulnificus* remain virulent, at least for a period of time, and could cause virulence and fatal infections following *in vivo* resuscitation. Zhang et al. (2015) reported that the UV can induce *E. coli* and *Pseudomonas aeruginosa* into VBNC state. Furthermore, they also demonstrated that both VBNC bacteria still highly expressed the virulence genes *gadA* and *oprL* and displayed pathogenicity. In Japan, the VBNC state of *E. coli* O157:H7 cells was found to be widespread in a natural freshwater environment and some caused gastroenteritis outbreak in salted salmon roe (Ohtomo and Saito, 2001; Zhao et al., 2017, 2018). This suggests that the VBNC pathogens could bring serious risks to food safety and public health.

Low temperature refrigeration is the most common way of food preservation. However, many research reported that low nutrient and low temperature are main causes of the induction of VBNC stages of pathogenic bacteria (Mizunoe et al., 2000; Besnard et al., 2002; Patrone et al., 2013). Therefore, it is necessary to know whether *E. coli* O157:H7 induced into a VBNC state at low temperature storage and can resuscitate and maintain their physiological characteristics. There will be of great significance for food safety. Thus, in this study, the standard strain *E. coli* O157:H7 (ATCC43895) was mainly used as the target strain, and the ability of the cells to enter the VBNC state under different environmental conditions was studied according to the possible environment of food storage in the low temperature. The metabolic activity of VBNC *E. coli* O157:H7 was detected by AODC and DVC methods. And the resuscitation methods on the VBNC cells induced by low temperature were also explored.

## Materials and Methods

### Bacterial Strains and Culture Conditions

Bacterial strains were stored at -80°C in Bacto^TM^ Tryptic Soy Broth (TSB; Becton Dickinson and Co., Sparks, MD, United States) containing 20% glycerol.

**FIGURE 1:**
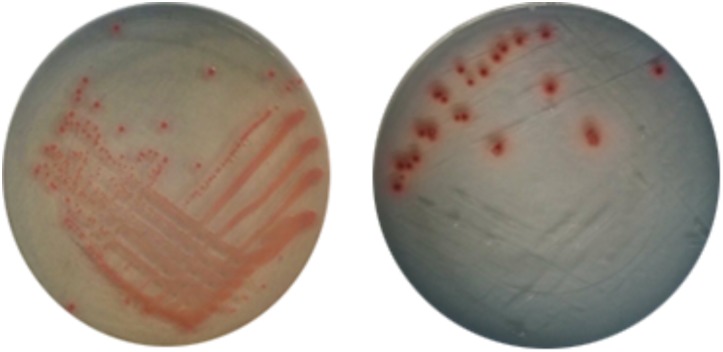
Bacterial colonies of *Escherichia coli* O157:H7 on chromogenic medium.

All the strains were cultured on chromogenic *E. coli* O157:H7 medium (Hopebio, Qingdao, China) for 24 h at 37°C. The bright red single colonies were used to identify presumptively the presence of *E. coli* O157:H7 (Figure 1). And then cells were incubated in Bacto^TM^ TSB at 37°C overnight with shaking (110 r/min).

### The Growth Curve of *E. coli* O157:H7

Overnight suspension of *E. coli* O157:H7 was diluted to 2% in 100 mL fresh Luria Bertani broth (LB; Invitrogen Co., Carlsbad, CA) then inoculated at 37°C in shaking bed with 190 r/min. Then the absorbance value (at 600 nm) of LB broth was determined every 0.5 h until 12 h. Duplicate experiments were performed, and final absorbance values were obtained as means of three determinations in each experiment. Therefore, the logarithmic growth phase of *E. coli* O157:H7 can be determined by growth curve.

### Low Temperature Induction of VBNC *E. coli* O157:H7

The overnight culture of *E. coli* O157:H7 bacterial suspension was inoculated into LB broth at 37°C, 190 r/min until the *E. coli* was cultured into mid-logarithmic phase. And then the initial number of CFU/mL about phase bacterial suspension was confirmed by plating serial decimal dilutions in sterilized saline water followed by overnight incubation at 37°C. Exponential phase cells were harvested by centrifugation at 8,000 × *g* for 5 min, washed three times with 0.85% (w/v) sterile saline solution remove nutrients. Cells were resuspended in the 0.85% (w/v) sterile saline solution at a final density of approximately 10^7^ CFU/mL. According to the environment and temperature of food preservation, the objective of this study was to induce *E. coli* O157:H7 under the conditions of physiological saline at -20°C, sterile distilled water at -20°C, LB broth at -20°C, sterile distilled water at 4°C, and LB broth at 4°C, respectively. Bacteria were counted every 4 days to determine their culturability. In order to avoid the influence of the repeated freezing and thawing by plate counting, we dispensed the mixed bacteria suspension 1 mL into several 1.5 mL tubes and placed at low temperature for induction.

### Total and Viable Counting of *E. coli* O157:H7

When less than 0.1 CFU/mL of the bacteria was culturable for three consecutive days, the cells were considered to be into the VBNC state. Total bacterial cell counts were determined by acridine orange direct count (AODC) according to Hobbie et al. (1977). 1 mL induction liquid was centrifuged at 4000 r/min for 5 min, washed three times with ultrapure water, and then diluted to a proper concentration gradient by plating serial decimal dilutions. An aliquot of 1 mL each sample was stained with 0.01% of acridine orange (Sigma, United States) in the dark. Before staining, the acridine orange solutions were filtered through a 0.2 μm pore size filter membrane (Millipore, United States). After 5 min 10 μL of suspension incubated with the dye mixture was captured by microfiltration through a 0.2 μm pore size black polycarbonate membrane filter and then applied to a slide with coverslip and viewed with a fluorescent inverted microscope (OLYMPUS, Japan). Viable counts of *E. coli* O157:H7 were determined by the direct viable count (DVC). One milliliter of each sample was centrifuged at 4000 r/min for 5 min, washed three times with ultrapure water. The pellets were incubated with final concentration of 0.025% yeast extract (Becton Dickinson and Co., Sparks, MD, United States) in the presence of 0.002% nalidixic acid (Biotium, United States) at 37°C for 12 h. After that, the incubated suspension with the dye mixture of *E. coli* O157:H7 were determined according to the above procedure of AODC. All the numbers of total and viable cell counts were obtained as means of counts visualized in 10 randomly selected microscopic fields (Besnard et al., 2000).

### Scanning Electron Microscopy for VBNC *E. coli* O157:H7

Cells of *E. coli* O157:H7 (ATCC 43895), in VBNC state for 3 days, in the mid-exponential phase, and the killed by boiled water for 5 min, were centrifuged at 8000 r/min for 5 min and washed three times with sterile phosphate-buffered saline (PBS, pH 7.4). The harvested bacteria pellets were placed in a fixative 0.2 mol/L phosphate buffer solution containing 2.5% (wt/vol.) glutaraldehyde for 4 h at 4°C. After the fixed completion, the samples were rinsed three times with additional PBS to remove fixative and were further dehydrated for 10 min in graded ethanol series solutions (30, 50, 70, 80, and 90%), and then rinsed in 100% ethanol at 20 min intervals for two times. At last, all the samples were replaced with 100% tert butanol for 15 min and dried in freeze-drying machine. For scanning electron microscopy (SEM) assay, the samples were coated with gold palladium for 90 s in an IB-3 vacuum evaporator coating apparatus (Eiko) and were then examined with scanning electron microscope (Hitachi Instruments Inc., Japan).

### Resuscitation of VBNC *E. coli* O157:H7

The resuscitate ability of any bacterium is critical, if the VBNC state is the real survival strategy. To investigate the recovery culturability processes of the bacterium, a series of independent experiments was conducted. In this study, we took four methods such as improving temperature up at 37°C directly, increasing temperature from 15°C to 37°C, adding Tween 80 with improving temperature at 37°C and adding nutrients with improving temperature at 37°C to explore the resuscitation of VBNC bacteria. In this work, samples containing no supplements and without any treatment were designated as controls. At the same time, all experiments were carried out in three replicates, and the recovered cells would be validated on *E. coli* O157:H7 chromogenic medium.

To determine whether recovery was due to true resuscitation or to regrowth of a few culturable cells of *E. coli* O157:H7 that remained in the microcosms, in this study, all the samples were diluted tenfold in sterilized distilled water as described previously studies (Wai et al., 2000; Zhang et al., 2015).

Resuscitation of the VBNC cells was performed by temperature upshift treatment to 37°C. Thawed suspension of VBNC bacteria was added to the sterile centrifuge tube and incubated at 37°C in a static state. All the samples were sampled 100 μL every 12 h to determine culturability by plating on TSA and incubated at 37°C for 24 h to count.

Gradient temperature recovery method: resuscitation of the VBNC cells was performed by increasing temperature from 15°C to 37°C. Thawed suspension of VBNC bacteria was added to the sterile centrifuge tube. Each sample was incubated at 15°C for 1 h, 20°C for 1 h, 25°C for 1 h, 30°C for 1 h, 37°C for 1 h, and colony counts were determined by plating on TSA in each incubation period.

Resuscitation of the VBNC cells was performed by adding Tween 80 with improving temperature at 37°C. Tween 80 (Sigma, St. Louis, MO, United States) was diluted with distilled water to desired concentrations and sterilized. Each sample added sterilized Tween 80 to make the final concentration was 8, 5, 3, 2, 1, 0.5% (w/v), respectively, and incubated at 37°C with shaking. Each sample of colonies was enumerated after 24 h of incubation.

Resuscitation of the VBNC cells was performed by improving temperature at 37°C in the 25% (w/v) sterilized yeast extract (Becton Dickinson and Co., Sparks, MD, United States) broth with shaking. After 24, 48, and 72 h incubated, 0.1 mL sample were spread on plate count agar. At same time, samples containing no supplements were designated as control.

## Results

### The Growth Curve of *E. coli* O157:H7

**FIGURE 2:**
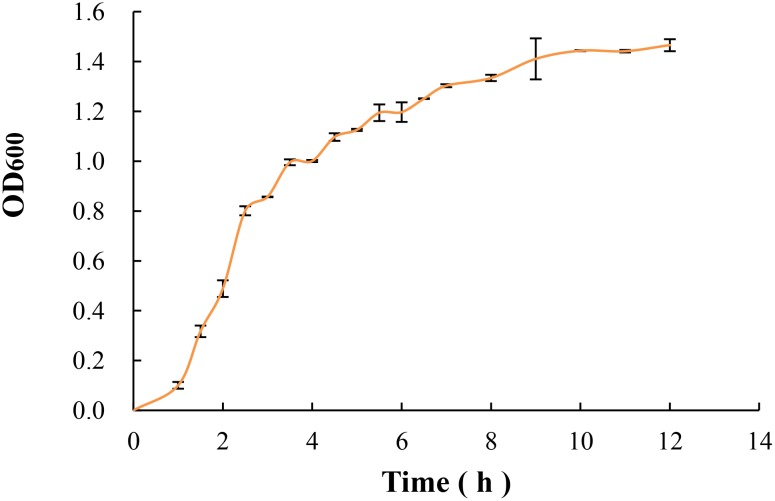
The growth curve of *E. coli* O157:H7 in LB medium at 37°C and 190 rpm.

As shown in Figure 2, the growth rate of *E. coli* O157:H7 grew rapidly in the geometric progression within 1 to 4 h. After 6 h, the growth rate slowed down and the absorbance value increased slowly. According to the relevant studies, the bacteria morphology and biological activity in the logarithmic phase are more typical, which are very sensitive to the external environment, and easier to access the VBNC state under certain induction conditions (Ayrapetyan et al., 2014; Zhou et al., 2015; Liu et al., 2017). Therefore, the bacteria incubated for 3 h in mid-exponential growth phase was selected as the study of subsequent induction in this study.

### The Culturable Cells of *E. coli* O157:H7 by Low Temperature

**FIGURE 3:**
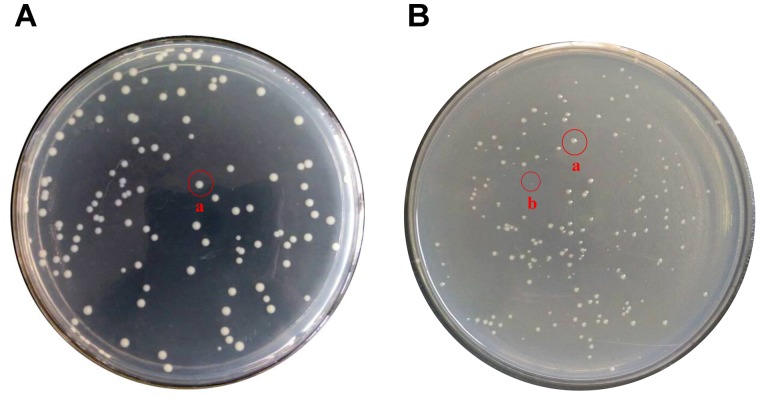
Changes of colony morphology of *E. coli* O157:H7 induced by low temperature. **(A)** The logarithmic phase of *E. coli* O157:H7 colonies on LB medium; **(B)** Colonies formed on LB medium of *E. coli* O157:H7 after a period of induction at a low temperature.

**FIGURE 4:**
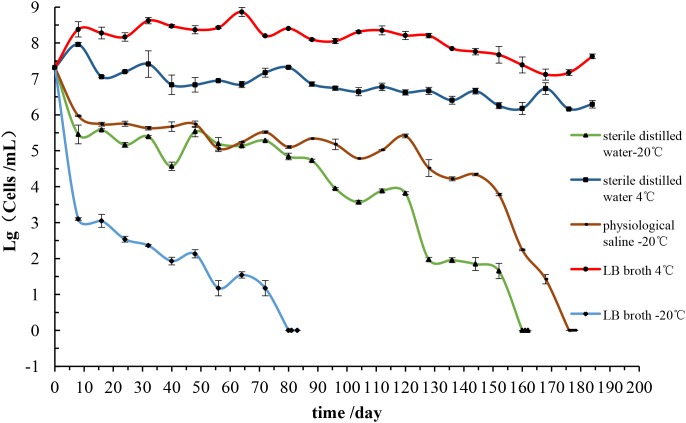
Culturable numbers of *E. coli* O157:H7 under different conditions.

In daily life, -20°C and 4°C are very important for food preservation. Therefore, it is of great practical significance to study the situation of *E. coli* O157:H7 under these two temperatures. Figures 3, 4 show the response of *E. coli* O157:H7 following its incubation in low temperature. Along with prolonging induction time, it could be seen that the colony morphology and culturable number were changed. Figure 3 shows the changes of their colony morphology in LB broth at -20°C, after incubated for 60 days. With the increase in induction time, the culture ability of most bacteria weakened, the colony morphology was smaller and formed invisible small colonies (As shown in Figure 3 labeled b). While those with strong ability to withstand low temperatures, the metabolism was still relatively stable and the colony morphology was close to the size of logarithmic phase (As shown in Figure 3 labeled a).

The response of the *E. coli* O157:H7 following its incubation at -20°C and 4°C were shown in Figure 4. The changes in culturable cell numbers of *E. coli* O157:H7 induced by low temperatures were investigated in this study. All the organisms maintained at 4°C showed the culturable colony counts increased, and the cells in LB broth rose to 10^8^ CFU/mL during the first day. Throughout 180 days, the culturable cells were a very slight decline at the end of the experiment and cells in LB broth and sterile distilled water at 4°C, the culturable colony counts remained near 10^8^ CFU/mL and 10^7^ CFU/mL, respectively. These results indicated that the *E. coli* O157:H7 remained strong metabolic viability at 4°C, thus it was not be stressed at this temperature (Vattakaven et al., 2006).

The culturability of *E. coli* O157:H7 following the prolonged storage in LB broth at -20°C decreased four logarithmic orders (from 10^7^ to 10^3^ CFU/mL) during the first 10 days. The initial inoculum of 2.1 × 10^7^ CFU/mL declined to below 0.1 CFU/mL to undetectable levels on the 80th day. Continuous cultivation for 3 days, the plate counts were always below 0.1 CFU/mL. At this stage, it could be considered that *E. coli* O157:H7 may enter to the VBNC state. However, the culturable number variation of *E. coli* O157:H7 in normal saline was similar to that in distilled water at -20°C. Both colony counts declined rapidly within first 10 days and then gradually decreased to undetectable levels at 176 and 160 days, respectively.

It demonstrates that the tolerance and viability of *E. coli* O157:H7 at -20°C are much less than that at 4°C. Beyond that, *E. coli* O157:H7 in eutrophic condition has poor tolerance to low temperature than in oligotrophic condition, and are more susceptible to loss of the culturability. This study also reveals that the *E. coli* O157:H7 in the physiological saline is more stable than in the distilled water environment at -20°C. It is well known that physiological saline has a balanced osmotic pressure on cells, which means that it has a certain protective effect on cells. Thus, the results may be due to the physiological saline that has a balancing effect on osmotic pressure of bacteria compared with sterile distilled water (Hemamalini et al., 2017).

However, the time required for the cells to enter the non-culturable state under this experimental condition exist difference from that of other studies, requiring a longer period of up to 180 days. This is sufficient to show that although in the same strain and the same intake conditions, the required time for cells to enter the non-culturable state is not consistent, which may be related to bacterial vaccination algebra, its source of physiological characteristics and resistance to the environment.

### Total and Viable Counting of *E. coli* O157:H7

**FIGURE 5:**
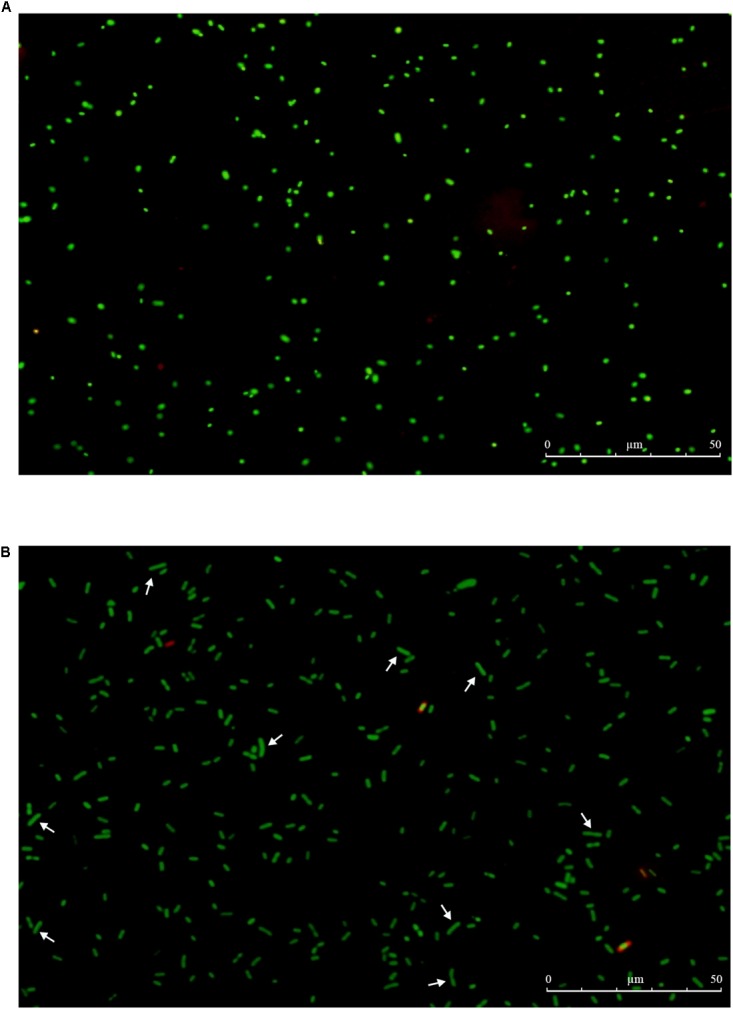
The total and viable number of *E. coli* O157 by fluorescence microscope. **(A)** VBNC cells by AODC; **(B)** VBNC cells by DVC.

When the initial inoculum of 2.1 × 10^7^ CFU/mL declined to undetectable levels or below 0.1 CFU/mL, total and viable cell counts were detected by AOAC method and by DVC method, respectively. The results showed that total and viable cell counts were 10^7^ cells/mL and 10^5^∼10^6^ cells/mL, respectively. At the same time, the non-culturable cells of *E. coli* O157:H7 exhibited fluorescing green, and the morphology were small coccoid cells by the AODC method under the fluorescent inverted microscope (Figure 5A). However, when the cells were detected by the DVC method, most of cells morphology had obvious growth and elongation compared to the cells by the AODC method detection (Figure 5B). The results confirmed that the elongated cells were defined as viable by the DVC method. Therefore, it also indicated that a large population of cells entered into the VBNC state.

### Morphological Changes of the VBNC *E. coli* O157:H7 by SEM

**FIGURE 6:**
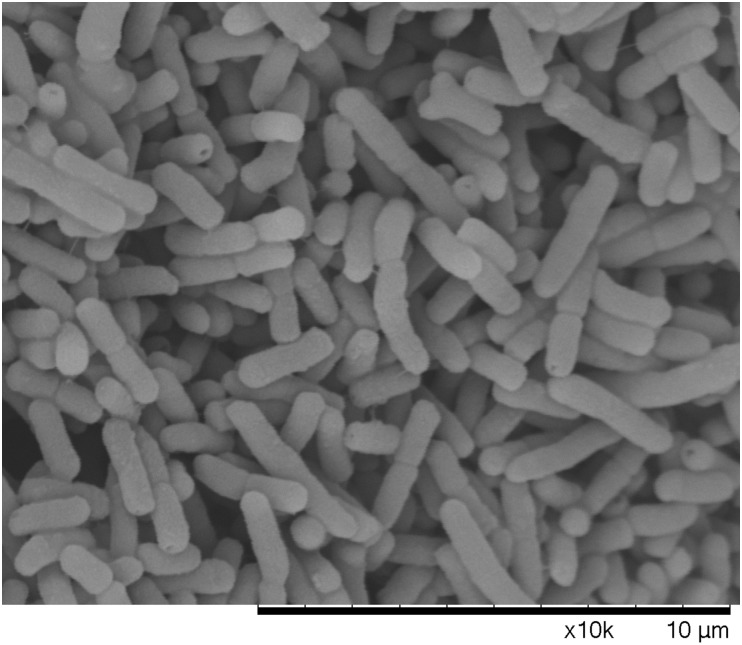
Logarithmic growth phase of *E. coli* O157 under the scanning electron microscope.

**FIGURE 7:**
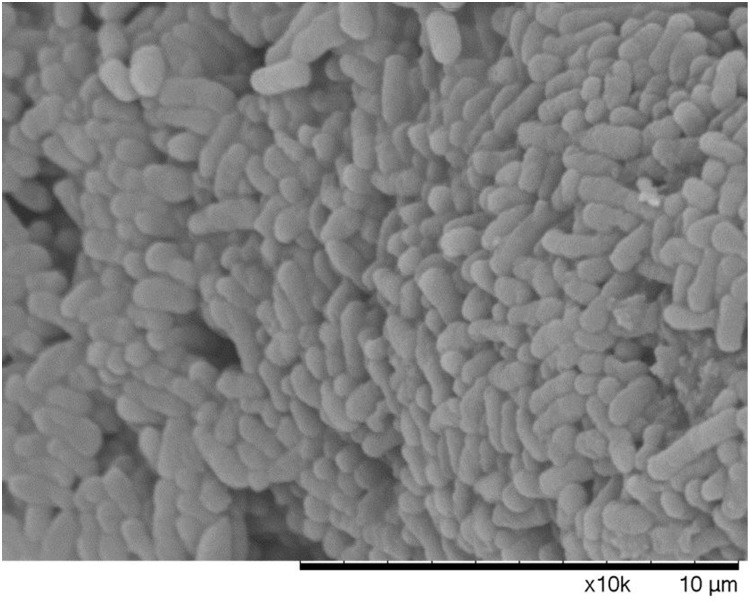
Dead *E. coli* O157 under the scanning electron microscope.

**FIGURE 8:**
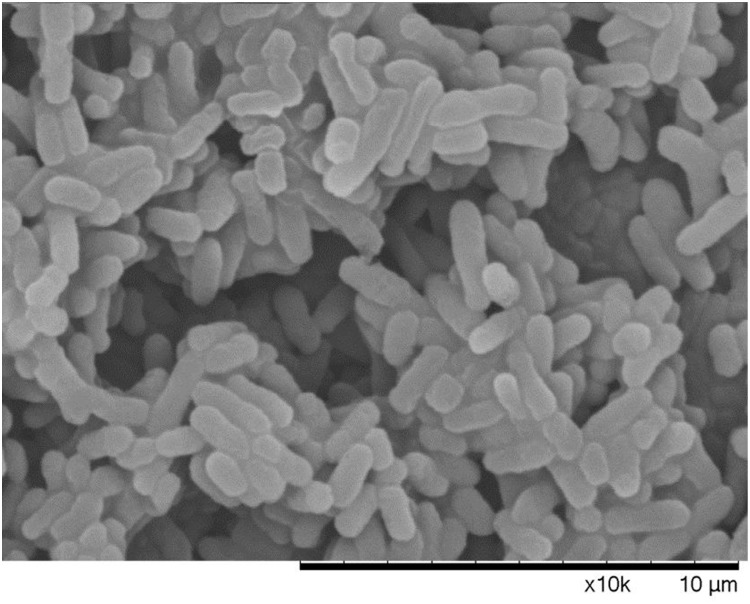
VBNC *E. coli* O157 under the scanning electron microscope.

The morphology of different states bacterial cells, mid-exponential-phase cells, boiling water bath for 5 min dead cells, and VBNC state, were observed by SEM. There was a clear difference among cells in the VBNC state, cells in mid-exponential-phase, and heating death state. It was found from Figure 6 that cells in mid-exponential-phase had the typical rod shape, smooth surface and a normal size with a uniform distribution. The surface of dead *E. coli* O157:H7 cells were relatively rough, aggregation, and even some cell surface damage (Figure 7). In the VBNC state, the cells changed from the typical rod shape to short rod and the cell size decreased, except that the cells were relatively rough, shrunken, the shape irregular, and some of them tended to be clustered (Figure 8). These results are consistent with those of Zhao et al. (2013), who reported that VBNC *E. coli* O157:H7 induced by high pressure CO_2_ and its characteristic.

### Resuscitation of *E. coli* O157:H7 in VBNC State and Validation

Viable but non-culturable cells were conducted in triplicate by different measures to examine the recovery potential and characteristics. When resuscitation of the VBNC cells was performed by increasing temperature from 15°C to 37°C, the results showed that the method was unable to induce resuscitation of *E. coli* O157:H7 and none colonies appeared on the plate (Table 1). Similarly, it was unable to resuscitate the VBNC *E. coli* O157:H7 by the methods of temperature upshift treatment to 37°C or adding nutrients with improving temperature at 37°C, even incubated for 1 month (data not shown). Thus, we performed experiments by adding Tween 80 with improving temperature at 37°C, and the results showed that *E. coli* O157:H7 in the VBNC state could resuscitate in the presence of Tween 80 with temperature upshift. The resuscitation results are listed in Table 2. After incubating with 5% Tween 80, VBNC *E. coli* O157:H7 cells produced a number of colonies with 7.2 × 10^2^ CFU/mL and 1.3 × 10^8^ CFU/mL on the plates at 24 and 48 h, respectively, while no colony was detected on the non-amended plates. The VBNC *E. coli* O157:H7 cells incubated with 3% Tween 80 for 48 h at 37°C, no colony was detected on the plates, but VBNC *E. coli* O157:H7 cells produced a number of colonies with 7.3 × 10^6^ CFU/mL after 72 h. Meanwhile, all the recovered cells were validated on *E. coli* O157:H7 chromogenic medium, and found that the colonies were bright red and consistent with the characteristics of the normal state of the cell, indicating that the resuscitation of VBNC bacteria restored its culture features. But, the bacteria cannot resuscitate with same conditions when *E. coli* O157:H7 were induced into VBNC state for 10 days. VBNC *E. coli* O157:H7 were incubated in other concentrations of Tween culture, but no colony was observed on the plate. At the same time, all the sterile colonies (-) shown in Table 2 were the result of the VBNC bacterial incubation time extended to 30 days. It also indicated that not all methods could effectively resuscitate VBNC bacteria.

**Table 1:** Effect of gradual warming on *E. coli* O157:H7.

Temperature (°C)	Culture time (h)	Bacteria count (CFU/mL)
15	1	-
20	1	-
25	1	-
30	1	-
35	1	-
37	1	-
37	12	-
37	24	-


*-, means sterile colonies.*

**Table 2:** Recovery by tween 80 for the bacteria induced by the low temperature.

Induction conditions	The final concentrations of Tween 80						Blank controls
		
	8%	5%	3%	2%	1%	0.5%	
LB broth	-	+	-	-	-	-	-
Physiological saline	-	-	+	-	-	-	-
Distilled water	-	-	-	-	-	-	-


*Tween concentration of 8, 5, 3, 2, 1, and 0.5% are accounted for the proportion of bacterial suspension. -, means sterile colonies. +, means colonies grow. All the sterile colonies (-) shown in this table were the result of the VBNC bacterial incubation time extended to 30 days.*

## Discussion

It is crucial to understand the occurrence and potential hazard of non-culturable foodborne pathogens for food safety. Low temperatures (-20°C and 4°C) are commonly used to preserve food products in our life. In this study, *E. coli* O157:H7 cells were induced to the VBNC state at -20°C and 4°C in different conditions. In the first 8 days, all the organisms maintained at 4°C showed the culturable colony counts increased especially for LB broth. We conjectured that *E. coli* O157:H7 of mid-exponential growth phase had strong growth and reproductive ability, a low temperature of 4°C did not inhibit its reproductive growth in a short time. The LB broth contains essential nutrients for growth and reproduction of *E. coli* O157:H7, thus the culturable colony counts increased especially. Meanwhile, Clavero and Beuchat (1996) reported that regardless of the pH and a(w) of TSB, survival of *E. coli* O157:H7 at 5°C was better than at 20°C or 30°C. The cells incubated at -20°C were induced to the VBNC state in three conditions (LB broth, distilled water, physiological saline), which needed 80, 160, and 176 days, respectively. These results are similar with Tian et al. (2013), which also reported that *E. coli* O157:H7 was induced to enter the VBNC state in LB broth at low temperatures (-18°C) for a shorter time than in physiological saline. While the reason for this result is not clear at the moment, we suspect that this may be related to the adaptability of bacteria in different conditions. However, the cells were incubated at 4°C about 190 days, culturable numbers were maintained at a bacterial concentration greater than 10^7^ CFU/mL. Our results were similar to those of Zhou et al. (2015), who reported that the culturable number of *E. coli* O157:H7 ATCC 43895 remain 10^7^ CFU/mL and failed to enter VBNC state at 4°C with limited nutrient. Compared with previous literatures, Masmoudi et al. (2010) showed that *S. aureus* spp. (exponential growth phase) were induced into the VBNC state after 210 days under filtered-sterilized seawater at 4°C. Rao et al. (2014) reported that *V. vulnificus* in artificial sea water at 4°C incubated into VBNC state need 70 days. Patrone et al. (2013) showed that the 10^8^ cells/mL of *C. jejuni* 241 and *C. jejuni* ATCC33291 in fresh water at 4°C were induced to VBNC state after 48 and 46 days, respectively. Therefore, these results suggested that the cells incubated at 4°C may spend more time for VBNC state formation than the cells incubated at -20°C or fail to enter this state until death. In addition, it was found that the ability and the speed of the VBNC state induction were mainly related to the incubation conditions and species of the cells and species. This study used fluorescence microscopy and scanning electron microscopy to observe the viability status and morphological changes in VBNC samples. It suggested that the VBNC state *E. coli* O157:H7 may exist in the qualified frozen foods detected by the national standard culture method and had threat to the food safety and also provided the basic data for the microbial detection standard. The non-culturable cells of *E. coli* O157:H7 were detected by the DVC method, most of cells morphology had obvious growth and elongation compared to the cells by the AODC method detection. It suggested that the VBNC state *E. coli* O157:H7 was still alive. Morphological and characteristics of VBNC cells were observed in this work. Compared with mid-exponential-phase cells, the cells entered into VBNC which morphological changed from the typical rod shape to short rod and the cell size decreased, except that the cells were relatively rough, shrunken, the shape irregular, and some of them tended to be clustered. This state was between logarithmic and dead cells and caused by bacteria to resist environmental pressure. And the morphological of VBNC cells were consistent with other researchers reported (Pawlowski et al., 2011; Zhao et al., 2013). In our study, the formation of *E. coli* O157:H7 VBNC is mainly induced by low temperatures, which are food preservation condition. Zhao et al. (2013) are mainly induced *E. coli* O157:H7 into the VBNC state by high pressure CO_2_ (HPCD), which is food processing conditions. It can be obtained that bacteria are subjected to different environmental stresses, the ability and the speed of the VBNC state induction were also different. At the same time, it also can indicate that the foodborne pathogens may enter the VBNC state during food processing techniques, such as high pressure, low temperature storage high temperature, and they have become a potential risk for food safety. If the VBNC state is a real strategy to survive the pressure of the environment, the resuscitate ability of any bacterium is critical. Therefore, we attempted variety of methods to resuscitate *E. coli* O157:H7. Our resuscitation experiments showed that *E. coli* O157:H7 in LB and physiological saline at -20°C induced VBNC state can resuscitate in 5% Tween 80 and 3% Tween 80, respectively, at 37°C, which has not been recognized previously. However, the VBNC states were not resuscitated by temperature upshift treatment, adding nutrients with improving temperature and gradient warming. There was clear difference between some previous literatures reported (Nilsson et al., 1991; Wong et al., 2004). But, adding Tween 80 at 37°C were used to resuscitate VBNC cells, the cells could not resuscitate again with same methods after 10 days, and the results were similar to Tian et al. (2013) reported. It was confirmed that there may be a mechanism to make VBNC state regain the ability to divide, i.e., resuscitation, otherwise dead end. And the resuscitation was also influenced by the VBNC state induction conditions and also associated with bacterial vaccination algebra, the source of their own physiological characteristics. It is worth emphasizing that failing of resuscitation does not mean that the cells cannot be resuscitated in principle before suitable methods have been found (Chen et al., 2018). VBNC *E. coli* O157:H7 may pose a potential health risk. The results of this study will help to better understand the health risks associated with VBNC bacteria.

## Conclusion

In this study, the initial inoculum of 2.1 × 10^7^ CFU/mL *E. coli* O157:H7 cells were induced into the VBNC state in normal saline, distilled water, LB broth at -20°C after 176, 160, 80 days, respectively. Through detecting VBNC cell by the DVC methods and AODC method and SEM, the *E. coli* O157:H7 VBNC cells still have metabolic activity and the cell morphology changed from the typical rod shape to short rod and the cell size decreased. VBNC *E. coli* O157:H7 cells could regain the ability to divide after adding Tween 80 at 37°C. This study indicates that *E. coli* O157:H7 can be induced into VBNC state with active metabolism by low temperature and also can resuscitate to fully culturable. The formatting of VBNC and the potential resuscitative strategy described in this study may have meaning to the risk assessment in food safety and pathogenic bacteria detection method.

## Author Contributions

XZ conceived and designed the experiments. XZ and CW performed the experiments and contributed to the writing of the manuscript.

## Conflict of Interest Statement

The authors declare that the research was conducted in the absence of any commercial or financial relationships that could be construed as a potential conflict of interest.
